# Navigating tumor angiogenesis: therapeutic perspectives and myeloid cell regulation mechanism

**DOI:** 10.1007/s10456-024-09913-z

**Published:** 2024-04-06

**Authors:** Fan Yang, Gloria Lee, Yi Fan

**Affiliations:** 1 Present Address: Department of Radiation Oncology, University of Pennsylvania, Philadelphia, PA 19104 USA; 2 Department of Obstetrics and Gynecology, Ren Ji Hospital, Shanghai Jiao Tong University School of Medicine, Shanghai, 200127 China; 3 Shanghai Key Laboratory of Gynecologic Oncology, Ren Ji Hospital, Shanghai Jiao Tong University School of Medicine, Shanghai, 200127 China

**Keywords:** Tumor angiogenesis, Anti-angiogenic therapy, Vessel normalization, Endothelial reprogramming, Macrophages, MDSCs, Neutrophils, Therapy resistance, Immunotherapy, Radiochemotherapy

## Abstract

Sustained angiogenesis stands as a hallmark of cancer. The intricate vascular tumor microenvironment fuels cancer progression and metastasis, fosters therapy resistance, and facilitates immune evasion. Therapeutic strategies targeting tumor vasculature have emerged as transformative for cancer treatment, encompassing anti-angiogenesis, vessel normalization, and endothelial reprogramming. Growing evidence suggests the dynamic regulation of tumor angiogenesis by infiltrating myeloid cells, such as macrophages, myeloid-derived suppressor cells (MDSCs), and neutrophils. Understanding these regulatory mechanisms is pivotal in paving the way for successful vasculature-targeted cancer treatments. Therapeutic interventions aimed to disrupt myeloid cell-mediated tumor angiogenesis may reshape tumor microenvironment and overcome tumor resistance to radio/chemotherapy and immunotherapy.

## Introduction

Angiogenesis, the formation of new blood vessels from pre-existing ones, is a hallmark of cancer. Tumor angiogenesis is a pivotal process that promotes cancer growth, progression, and metastasis and induces therapy resistance [1]. Over the past few decades, considerable efforts have been directed towards understanding the molecular and cellular mechanisms that underlie tumor angiogenesis. These lead to the development of promising anti-angiogenic therapeutic strategies that aim to inhibit overgrowth and sprouting of tumor endothelial cells (ECs). Beyond the traditional anti-angiogenic concept that focuses on vessel delivery function, recent advances have revealed that the interactions between the tumor vasculature and the immune system are critical for regulation of tumor vascularity and immunity [2, 3]. The tumor microenvironment (TME) is a complex milieu composed of various non-neoplastic cell types, including ECs, stromal cells, and a diverse array of immune cells. The dynamic interplay between these cellular components in the vascular TME has significant implications for tumor development, immune evasion and the efficacy of cancer therapies, particularly immunotherapies. Therefore, development of efficient therapeutic strategies that reprogram the vascular TME will offer exciting opportunities for cytotoxic radio/chemotherapy and T cell-based immunotherapy.

In this review, we discuss the emerging strategies for tumor vasculature-targeting therapy. We provide a comprehensive overview of the complex regulation of tumor angiogenesis by myeloid cells, including macrophages, myeloid-derived suppressor cells (MDSCs), and neutrophils within the TME. We discuss the impact of these immune cells on tumor angiogenesis. We highlight that myeloid cells interact with ECs to regulate tumor angiogenesis and create a specialized niche that induces immune evasion and promotes tumor growth, providing crucial targets for vasculature-targeting therapy. These approaches may have the potential to revolutionize cancer treatment, paving the way for more effective therapeutic strategies.

## Tumor angiogenesis

### Basic principle of aberrant tumor angiogenesis

Tumor angiogenesis is fundamental to cancer progression, metastasis, and therapy resistance. Tumor angiogenesis refers to the pathophysiological process where new blood vessels sprout from pre-existing ones to supply nutrients, oxygen, and cellular network for tumor growth [1, 4–7]. The intricate network of blood vessels also allows cancer cells to infiltrate the bloodstream and disseminate throughout the body, giving rise to metastasis. These collectively suggest anti-angiogenic therapy, a treatment that aims to inhibit EC overgrowth and sprouting, as a promising strategy for cancer treatment. Notably, the newly formed vessels are structurally and functionally abnormal—they are tortuous and leaky with a disorganized, haphazard architecture. This abnormal vasculature leads to a chaotic blood flow, which creates a heterogeneously hypoxic tumor microenvironment [8]. Such hostile conditions can foster cancer cells that are more aggressive and therapy resistant, further promoting tumor growth and metastasis. Furthermore, the abnormal vessels also form a barrier to the effective delivery of drugs to the tumor, thereby contributing to therapy resistance [9]. Vessel normalization has, therefore, joined anti-angiogenic treatment as promising strategies for solid tumor treatment.

**Fig. 1:**
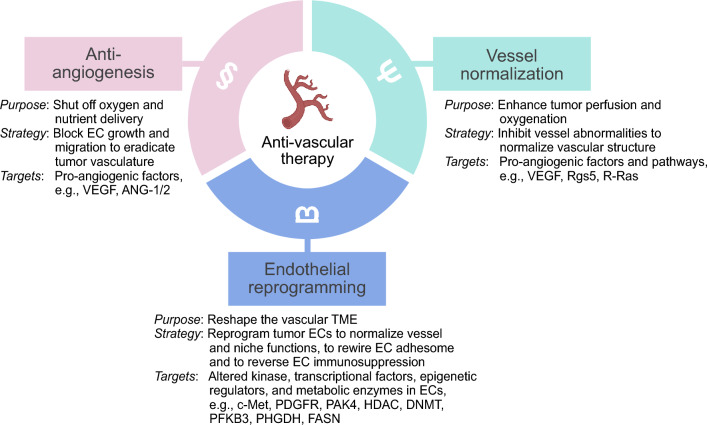
Therapeutic strategies for vasculature-targeting anti-cancer treatment. Therapeutic strategies targeting tumor vasculature have emerged as transformative for cancer treatment, encompassing anti-angiogenesis, vessel normalization, and endothelial reprogramming

Tumor angiogenesis is a complex process, subject to regulation by a balance between pro-angiogenic and anti-angiogenic factors within a solid tumor [10, 11]. When the equilibrium tilts toward pro-angiogenic factors, ECs are stimulated to proliferate and migrate towards the tumor, forming new blood vessels. The presence of excessive pro-angiogenic factors further stimulates vascular abnormalities [12, 13]. This imbalance drives both neovascularization and vascular aberrancy, serving as a critical therapeutic target for vessel normalization and cancer treatment. Finally, tumor ECs undergo genetic and metabolic alteration to acquire pro-tumor phenotypes including aberrant vessel structure and function, a rewired adhesome that reduces lymphocyte attachment, and local release of immunosuppressive molecules. Thus, anti-angiogenesis, vessel normalization, and endothelial programming serve as promising strategies for vasculature-targeting approaches for cancer treatment (Fig. 1).

### Anti-angiogenic therapy

#### Anti-VEGF/VEGFR

Anti-angiogenic therapy represents a promising strategy for cancer treatment by inhibiting the formation of new blood vessels that nourish tumors, thus depriving them of essential oxygen and nutrients for growth. Numerous anti-angiogenic agents targeting pro-angiogenic factors, such as vascular endothelial growth factor (VEGF, i.e., VEGF-A), fibroblast growth factors (FGF), and epidermal growth factor (EGF), have been extensively explored [14–16]. Among the most widely used anti-angiogenic agents are the monoclonal antibodies and tyrosine kinase inhibitors (TKIs) that target VEGF and the VEGF downstream kinases, respectively. VEGF plays a crucial role in both physiological and pathological angiogenesis [4]. In tumors, overexpression of VEGF, mainly driven by hypoxia-inducible factor (HIF)-1α, promotes abnormal blood vessel growth and acts as a vascular permeability factor [17]. VEGF usually binds to the tyrosine kinase receptor VEGFR2, in collaboration with neuropilin-1 and VEGFR3, and interacts with other modulating pathways such as Notch, angiopoietin/Tie2, and ephrin/Eph to facilitate vessel growth [18–22]. A number of anti-angiogenic agents have been approved by FDA for treating cancer, highlighting their role in current oncology therapeutics. For instance, bevacizumab (Avastin), a humanized monoclonal antibody that blocks VEGF, is a notable example of an anti-angiogenic agent demonstrating anti-tumor results in colon and kidney cancers [23]. Additionally, small-molecule pharmacological inhibitors of VEGF receptor tyrosine kinase, such as sunitinib and sorafenib, also offer a promising opportunity to cancer therapy [24, 25]. However, the overall efficacy of these anti-angiogenic therapies is often limited and does not produce long-term benefits in patients with most other cancer types, such as glioblastoma [26–30].Both intrinsic and acquired mechanisms contribute to tumor resistance to anti-angiogenic therapy, driven by the existence of redundant angiogenic pathways and the adaptive mechanisms that lead tumor cells to survive an avascular and hypoxic TME, respectively [6, 28].

#### Inhibition of vascular maturation

Inhibition of vascular maturation, a key aspect of functional vascularity, represents another therapeutic strategy for cancer. The EC growth factor signaling pathways composed of angiopoietin (Ang)-1/2 and their receptor, Tie2, play a critical role in this process. Ang-1, mainly secreted by pericytes and smooth muscle cells, promotes vascular remodeling and stabilization. Ang-1 overexpression is often observed in tumor vasculature, which enhances EC proliferation and pericyte-mediated vascular maturation, and increased vascular functions intensify the malignancy of various cancers [31]. Conversely, Ang-2 can induce angiogenesis and destabilize vasculature by binding to Tie2 and integrin receptors [32]. Given the role of angiopoietins in vascular biology, antibodies targeting these angiopoietins and dual inhibitors of Ang-2 and VEGF show promising results in various malignancies [20].

#### Targeting the development pathways of ECs

Tumor angiogenesis is tightly controlled by EC differentiation and growth. This process can be triggered by hypoxia [33], Notch [34], and Wnt signaling pathways [35]. Hypoxia, typically resulting from rapid tumor growth and disordered vasculature, initiates a survival response within tumors. To survive under these extreme conditions, tumors employ a host of mechanisms, primarily the activation of HIFs that induce transcription of hypoxia-adaptive metabolic enzyme and VEGF [36, 37]. Moreover, hypoxia can stimulate activation of mTOR, PI3K, and AKT through post-translational modifications of these proteins [38], which are central to EC metabolism, survival, and motility regulation in response to nutrient and oxygen depletion. Notch signaling emerges as a pivotal player in the orchestration of vessel sprouting, branching, and maturation. Aberrations in Notch signaling have been linked to tumor angiogenesis, positioning the Notch pathway as a potential target for anti-angiogenic cancer therapies [34]. A recent study on DII4-induced Notch signaling in EC growth and development shows that antibodies against Dll4 and VEGF had strikingly different effects on tumor blood vessels [39], suggesting differing mechanisms underlying Notch- and VEGF-mediated tumor angiogenesis. Dll4-driven Notch signaling appeared vital during active blood vessel formation, but less so for maintaining normal vessels [39]. Beside its established role in developmental angiogenesis and vascular differentiation, Wnt pathway has been implicated in tumor angiogenesis. The interaction between Wnt and Frizzled receptors activates varied signaling outcomes in both canonical and non-canonical pathways, contributing to regulation of EC functions. For example, canonical Wnt-frizzled signaling produces a β-catenin/Lef/TCF complex which triggers brain angiogenesis during development [40]. In the context of GBM, activation of Wnt/β-catenin signaling in ECs is associated with chemoresistance [41], highlighting a potential target in GBM treatment. On the other hand, in the non-canonical pathway, Ca^2+^/calmodulin-dependent protein kinase II (CAMKII) activation influences ventral cell fate [42], and other signaling cascades like JNK and Daam-1 drive EC proliferation and maintain cellular architecture [43–45].

Overall, these findings highlight multiple regulatory mechanisms, mediated through hypoxia, VEGF, Notch, and Wnt, for EC proliferation, migration, and differentiation during tumor angiogenesis. Understanding their dysregulation in cancer may help develop new targets for anti-angiogenic therapies.

### Vessel normalization therapy

Anti-angiogenic therapy can adversely enhance tumor hypoxia and reduce drug delivery, resulting from destroyed tumor vasculature, leading to increased resistance of tumors to radio/chemotherapy and targeted molecular therapy. Considering structurally and functionally abnormal vascularity in cancer, i.e., tortuous, leaky vasculature due to dysfunctional EC sprouting and overgrowth, a different strategy, namely, vessel normalization, that aims to restore normal vessel function, has been exploited [11]. This could be achieved by re-balancing the pro- and anti-angiogenic factors presented in the TME, with reduced hypoxia, improved perfusion allowing for proper drug delivery, and enhanced immune cell infiltration [12, 13]. Previous preclinical studies show that vessel normalizing doses of anti-VEGF treatment improve T cell infiltration and enhance immunotherapy [46, 47], due to enhanced vessel delivery and reduced intratumoral hypoxia. Moreover, a recent clinical trial shows promising results for combining anti-VEGF bevacizumab with immune checkpoint blockade in liver cancer treatment [29].

Additional therapeutic strategies for vessel normalization include decreasing vascular leakiness, enhancing the structural integrity, increasing perfusion, and adding angiostatic factors, with multiple targets identified. For example, targeting regulator of G protein signaling 5 (Rgs5) protein leads to more typical vessel morphology and function in tumors, without reducing vessel density [48]. Inhibiting L1CAM, a neural adhesion protein in tumor ECs, results in pruning and fortification of vessels, thereby reducing tumor growth and metastases [49]. Inhibition of neuronal nitric oxide synthase (nNOS) in cancer cells restores proper NO gradients, leading to denser and more effective vessels for oxygen and drug delivery [50]. Restoring semaphorin-3A (SEMA3A) initially prunes immature vessel, and long-term application increases vessel maturation [51]. Activation of R-Ras or lysophosphatidic acid (LPA) in ECs promotes vascular normalization [52, 53]. Chloroquine, known for its antimalarial properties, also plays a role in vessel normalization through endosomal Notch1 trafficking and signaling in ECs [54]. Activation of transient receptor potential vanilloid-4 (TRPV4) in tumor ECs restores normal mechanosensitivity and increases drug delivery [55]. Further strategies include using thrombospondin-1 (TSP-1), an endogenous antiangiogenic factor, to normalize vessels, enhance drug delivery, and increase the effectiveness of treatments like cisplatin [56].

Another innovative approach in vessel normalization involves modulating various cells within the perivascular niche. For instance, eribulin, a chemotherapy agent, regulates endothelial-pericyte interactions to fortify vessels and improve treatment outcomes [57]. Desmoplasia, characterized by fibrotic tissue growth, impairs vascular function by compressing vessels [13]. Therefore, normalizing the extracellular matrix (ECM) is crucial, as it can improve both vascular function and treatment outcomes. Strategies targeting cancer-associated fibroblasts and the extracellular matrix, such as inhibiting TGF-β [58] or sonic hedgehog pathways [59], and using Nab-paclitaxel [60], show promise in reducing vessel compression. Additionally, altering metabolic pathways in pro-tumor macrophages leads to the formation of well-organized and fortified vessels, thereby enhancing oxygen delivery [61]. Antitumor CD4^+^ T cells also play a role in normalizing vessels by modulating angiogenic gene expression in tumors [62]. Inhibiting VEGF expression from these T cells further suggests their role in promoting abnormal tumor vessel phenotypes [63]. These findings highlight the complex interactions among ECs, other cells, and ECM in the TME, which may induce vessel abnormalities. Understanding and targeting these interactions can normalize tumor vasculature and improve cancer therapy outcomes.

Although these strategies hold promise, the benefits of vessel normalization monotherapy have often been small and transient. For example, administration of low-dose bevacizumab to control excessive EC growth has been a central method used in vessel normalization. However, vessel normalization anti-VEGF therapies often lead to a transient window that is potentially open for additional therapies, after which tumors become resistant [64, 65]. Furthermore, the timing and dosing of vessel normalization therapy needs to be further optimized when combined with immunotherapies and other conventional cytotoxic therapies, as tumor immunogenicity and vascularity change over tumor development and treatment exposure [6, 13].

### Endothelial reprogramming therapy

EC plasticity has been well characterized during embryogenesis [66, 67]. In pathological settings including cardiac, renal, and liver fibrosis, ossifying myositis, pulmonary hypertension, and cerebral cavernous malformation, ECs can take endothelial mesenchymal transition (Endo-MT) de novo to generate fibroblasts and stem-like cells [68–70]. Notably, cell plasticity plays a central role in the EC transcriptomic alteration and aberrant vascular phenotypes in cancer [71, 72]. As an alternative process to angiogenesis and vascular abnormality driven by pro-angiogenic factor-induced vessel sprouting and outgrowth, ECs retain key endothelial functions but undergo cell plasticity-mediated genetic reprogramming to induce aberrant vascularity in the tumor microenvironment. For example, ECs acquire partial Endo-MT, also known as endothelial transformation, to promote their ability to proliferate, migrate and secrete [71–73]. These transformed ECs, unlike normal ECs, take transcriptomic alteration to drive distinct behaviors due to the influence of the TME, forming an abnormal architecture of tumor vasculature. This leads to poor perfusion and hypoxia within the TME, which fosters the selection of more aggressive, treatment-resistant cancer cells [74], and creates a physical barrier that shields tumor cells from immune cell attack and impedes the delivery of chemotherapeutic drugs, thereby inducing tumor resistance to chemo/radiotherapy and immunotherapy [7, 28]. The strategy for genetic reprogramming of tumor ECs, e.g., by targeting EC plasticity, aims to normalize these cells by reversing their abnormal traits of gene expression, making the vasculature resemble the normal one in structure and function, and, therefore, may eventually improve the efficacy of cytotoxic treatment and immunotherapy approaches [71]. In addition to transcriptomic alteration, tumor ECs also undergo metabolic changes in the TME [75]. Metabolic switches in tumor ECs are driven by genetic and epigenetic alteration of metabolism-associated genes in response to the cues in the TME, such as hypoxia. The adaptively rewired metabolism fosters EC survival and growth in the TME, contributing to aberrant tumor angiogenesis. Metabolic reprogramming of tumor ECs, therefore, serves as an additional strategy for vasculature-targeting cancer therapy [75].

#### Genetic reprogramming of ECs

The approach of genetic reprogramming of tumor ECs is initially termed as vascular de-transformation therapy, emphasizing its main target on EC plasticity [71]. Genetic reprogramming of tumor ECs would be expected to induce the formation of a stable, functionally normal, and structurally orderly vasculature, which reduces tumor hypoxia, improves drug delivery, and alleviates immunosuppression, thereby enhancing anti-tumor immune responses and the efficacy of other therapies [72]. Several strategies have been exploited for the genetic reprogramming of tumor ECs. HGF/c-Met is identified as a critical regulator of Endo-MT in cancer [73]. EC-specific c-Met knockout inhibits EC plasticity, reduces vascular aberrancy, and sensitizes tumor to chemotherapy [73]. Moreover, c-Met-mediated activation of Wnt signaling drives transformation of ECs into mesenchymal stem cell-like cells, leading to multidrug resistance in ECs and tumor chemoresistance [41]. Furthermore, platelet-derived growth factor (PDGF)-mediated EC plasticity controls VEGFR2 expression through Snail, which contributes to tumor resistance to anti-VEGF treatment [9]. Based on these results, combination of anti-PDGFR and anti-VEGFR therapy was explored in tumor, which shows promising synergistic anti-tumor effects [9]. A more recent whole kinome-wide screen identifies that p21-activating kinase 4 (PAK4) is a key driver of Endo-MT in cancer [76]. Inactivation of PAK4 reprograms transcriptome in ECs and normalizes tumor vasculature. Notably, genetic and pharmacological ablation of PAK4 in ECs reshapes the immune landscape within the TME, improving T-cell infiltration and sensitizing tumor to CAR-T cell therapy [76]. Furthermore, several additional targets have been identified for endothelial reprogramming, including ERG, Myct1, and Lrg1. Forced expression of ERG, a transcription factor essential for endothelial homeostasis, restores the angiogenic balance in tumor ECs, thereby inhibiting tumor growth and vascular abnormalities [77]. Interestingly, Myct1, a downstream protein of ETV2 and Myc, which is primarily expressed in ECs, plays a crucial role in mesenchymal-like transcriptional activation. Myct1 deficiency in mouse tumor models decreases angiogenesis and increases antitumor immunity, thereby limiting tumor growth [78]. Lrg1 is exclusively expressed on tumor ECs rather than normal ECs or pericytes. Deletion or antibody-based neutralization of Lrg1 results in vessel normalization and promotes the TME toward an anti-tumor, immune-active state, enhancing the efficacy of various cancer therapies [79].

In addition to structural abnormalities, tumor vasculature is also characterized by altered EC adhesiveness. Tumor ECs undergo genetic alteration, often with downregulated adhesion proteins, such as intercellular adhesion molecule 1 (ICAM-1) and vascular cell adhesion molecule 1 (VCAM-1) that are necessary for immune cell attachment and extravasation [75, 80]. This leads to less T cell attachment to the endothelium, inhibiting T cell infiltration and contributing to tumor immune evasion. It is tempting to speculate that mesenchymal-like activation drives this dysfunctional adhesion in tumor ECs, induced by epithelial-mesenchymal transition (EMT)-associated transcriptional repressors including Snail, Slug, Twist-1/-2, and Zeb-1/-2. As such, inhibition of PAK4 reduces expression of Slug and Zeb-1, upregulating expression of VCAM-1 and Claudin-14 in tumor ECs, which eventually enhances T cell adhesion and improves CAR T cell immunotherapy [76]. Together, these findings underscore the potential of genetic reprogramming of tumor ECs as a promising approach for cancer treatment.

#### Epigenetic reprogramming of ECs

Epigenetic reprogramming in ECs represents another promising strategy for targeting tumor angiogenesis, considering tumor ECs undergo substantial epigenetic alterations to modulate their functionality in cancer. Acetylation of histone H3 has been well characterized in tumor ECs, which epigenetically regulates the expression of key genes essential for EC function and angiogenesis, including CLU, FBN1, TSPAN2, and ICAM1 [81]. The activity of histone deacetylases (HDACs), especially HDAC1, is central to this process: they regulate MMP14 and VCAM-1 expression, driving EC growth and the formation of vascular structures [82, 83]. Inhibitors targeting HDACs, such as trichostatin A (TSA) and suberoylanilide hydroxamic acid (SAHA), hold promise in anti-angiogenesis therapy, as they modulate the transcription of several crucial pro-angiogenic signaling components, including receptors VEGFR1 and VEGFR2 [84], HIF-1α, and VEGF [85]. HDAC inhibitors not only exhibit anti-angiogenic properties across various cancer types but also enhance leukocyte adherence and movement within tumor vessels, primarily through the upregulation of ICAM-1 [86], underscoring their potential to boost the effectiveness of immunotherapy. Moreover, histone methylation is also critical for tumor angiogenesis. EZH2, a key histone methyltransferase, reduces trimethylation of histone H3 at lysine 27 (H3K27me3), a repressive epigenetic mark, during Endo-MT induced by IL-1β and TGF-β2 [87]. Conversely, JMJD2B, a histone demethylase,epigenetically modulates Endo-MT by promoting repressive H3K9me3 occurring at the promoters of mesenchymal and TGF-β signaling genes, such as calponin (CNN1), AKT serine/threonine kinase 3 (AKT3), and sulfatase 1 (SULF1) [88].

Beyond histone modifications, DNA methylation significantly influences the behavior of tumor ECs and, consequently, the immune profiles as well. For instance, deletion of DNA methyltransferase 1 (DNMT1) in ECs inhibits tumor growth and reshapes the immune environment, due to the increased expression of cytokines, chemokines, cell adhesion molecules in ECs, such as Cxcl9 and Cxcl10 that are crucial for infiltration of CD8^+^ T cells into the tumor [89]. DNMT1 silencing in ECs also enhances the expression of IL-33, Ccl21, and Ccl19 that are critical for neogenesis of high endothelial venule (HEV), a specialized postcapillary venule adapted for lymphocyte trafficking. Moreover, DNMT inhibitor treatment boosts leukocyte infiltration into tumors by upregulating ICAM1 expression in ECs [86]. Interestingly, proangiogenic factor FGF2 promotes ERK-mediated DNMT1 phosphorylation and nuclear translocation to repress Cxcl9 and Cxcl10 transcription [89], suggesting feedback loops that regulate angiogenic pathway activation and epigenetic regulation.

In summary, recent studies identifying an intricate network of epigenetic regulation in ECs during tumor angiogenesis provide profound insights into the mechanisms driving epigentic regulation of EC functions, and opens new avenues for developing therapeutic strategies targeting these epigenetic alterations to inhibit tumor growth and enhance immunotherapy outcomes.

#### Metabolic reprogramming of ECs

Given metabolic adaptation is required for cell proliferation and migration, such as EC outgrowth and sprouting, targeting endothelial metabolism has emerged as a promising strategy for modulating tumor angiogenesis [75, 90–95]. This strategy may not only rewire tumor vasculature by targeting EC sprouting, but also recondition the metabolic TME as it changes the EC-derived metabolites that are locally released. A key regulatory pathway of endothelial metabolism is glycolysis, a process critical for EC survival and proliferation in the hypoxic TME, as it generates necessary energy and metabolites anaerobically. For instance, disruption of glycolysis via PFKFB3 inhibition stabilizes the vascular barrier by improving pericyte adhesion, reduces metastasis, and enhances the efficacy of cancer chemotherapy [90, 95]. Furthermore, decreasing aerobic glycolysis in tumor ECs reduces vascular abnormalities, increases T cell infiltration, and overcomes tumor resistance to immunotherapy [96]. Notably, PHGDH, which diverts glycolysis into a specific serine biosynthetic pathway, promotes aberrant tumor angiogenesis, through its role in regulating nucleotide synthesis and maintaining the redox balance essential for endothelial proliferation [94]. Endothelial metabolism also contributes to the immunosuppressive TME by providing immunomodulatory metabolites produced by the vascular niche. As such, inhibition of serine metabolism in tumors ECs reduces their production of lactate and 2-hydroxyglutarate, two immunosuppressants in the TME, leading to activation of anti-tumor immunity [94]. Beyond glucose metabolism, other metabolic pathways in ECs are also being explored as therapeutic targets. Loss of endothelial Adrb2, which encodes the β2-adrenergic receptor, leads to angiogenesis inhibition through oxidative phosphorylation [97]. Similarly, disrupting fatty acid metabolism in ECs, as evidenced by that knockdown of fatty acid synthase (FASN) and the loss of CPT1A, a critical enzyme in fatty acid oxidation (FAO), limit vessel sprouting and proliferation through mTOR signaling and nucleotide synthesis, indicating the role of lipid metabolism in maintaining the physical structure of tumor vessels [91, 92]. Additionally, restricting glutamine metabolism through glutaminase 1 (GLS1) impairs vessel sprouting due to disrupted EC proliferation and migration [93], highlighting the importance of glutamine in sustaining macromolecule production necessary for angiogenesis. Collectively, the metabolic processes within ECs are fundamental not just for their energy and biosynthetic needs but also play a pivotal role in maintaining the structural and functional integrity of blood vessels in the TME. Understanding of these regulatory pathways offer key insights into how blood vessels adapt and grow in the TME, opening up new possibilities for targeted therapies aimed at modulating tumor angiogenesis.

In summary, genetic, epigenetic and metabolic reprogramming of tumor ECs represent promising advances in vasculature-targeting therapy, with the potential to improve the efficacy of conventional cytotoxic treatments and immunotherapies [75]. There are potential drugs that may be tested for endothelial reprogramming therapy (Table 1). In addition, a number of clinical trials are currently undergoing to evaluate the synergistic effects of combining conventional anti-angiogenic agents, such as Bevacizumab and axitinib, with immunotherapies, aiming to enhance treatment efficacy and patient outcomes (Table 2).

**Table 1:** Potential drugs for endothelial reprogramming therapy

Name	Brand Name	Mechanism of action	Status
Ficlatuzumab/AV-299	N/A	Monoclonal antibody against HGF	Under clinical trials
YYB101	N/A	Monoclonal antibody against HGF	Under clinical trials
Cabozantinib	Cometriq, Cabometyx	Inhibitor of c-Met (and VEGFR2, AXL, and RET)	FDA-approved for medullary thyroid cancer, kidney cancer
Olaratumab	Lartruvo	Monoclonal antibody against PDGFRα	FDA-approved for soft-tissue sarcoma (STS)
Ripretinib	Qinlock	Inhibitor of PDGFRα (and KIT)	FDA-approved for advanced gastrointestinal stromal tumor (GIST)
Sunitinib	Sutent	Inhibitor of PDGFRs (VEGFRs and KIT)	FDA-approved for renal cell carcinoma (RCC), pancreatic cancer, and imatinib-resistant gastrointestinal stromal tumor (GIST)
KPT-9274	N/A	Inhibitor of PAK4 (and NAMPT)	Under clinical trials
Panobinostat/ LBH589	Farydak	HDAC inhibitor	FDA-approved for multiple myeloma
Vorinostat/SAHA	Zolinza	HDAC inhibitor	FDA-approved for cutaneous T-cell lymphoma (CTCL)
Belinostat/ PXD101	Beleodaq	HDAC inhibitor	FDA-approved for peripheral T-cell lymphoma
Romidepsin/ FK228	Istodax	HDAC inhibitor	FDA-approved for cutaneous T-cell lymphoma (CTCL) and other peripheral T-cell lymphomas (PTCLs)
Azacitidine	Vidaza	DNMT inhibitor	FDA-approved for myelodysplastic syndrome, myeloid leukemia, and juvenile myelomonocytic leukemia
Decitabine	Dacogen	DNMT inhibitor	FDA-approved for myelodysplastic syndromes and acute myeloid leukemia (AML)
TVB-2640	N/A	FASN inhibitor	Under clinical trials
Etomoxir	N/A	CPT1A inhibitor	Under clinical trials
IACS-6274	N/A	GLS1 inhibitor	Under clinical trials

**Table 2:** Clinical trials evaluating combination of anti-angiogenic and immunotherapies

Clinical Trial Identifier	Phase	Status	Angiogenic Therapy	Immunotherapy	Other Drugs	Cancer
NCT05488522	I	Recruiting	Bevacizumab	Atezolizumab	Stereotactic body radiotherapy (SBRT)	Advanced hepatocellular carcinoma (HCC)
NCT02873195	II	Active	Bevacizumab	Atezolizumab	Capecitabine	Refractory metastatic colorectal cancer
NCT04356729	II	Recruiting	Bevacizumab	Atezolizumab	–	Unresectable or metastatic stage II or IV cutaneous melanoma
NCT03762018	III	Active	Bevacizumab	Atezolizumab	Standard chemotherapy	Malignant pleural mesothelioma
NCT03074513	II	Active	Bevacizumab	Atezolizumab	–	Rare solid tumors
NCT02210117	I	Active	Bevacizumab	Nivolumab, Ipilimumab	–	Resectable metastatic kidney cancer
NCT06083844	II	Recruiting	Bevacizumab	Pembrolizumab	Low-dose cyclophosphamide	High grade ovarian cancer with minimal residual disease after frontline treatment
NCT03175432	II	Active	Bevacizumab	Atezolizumab	Cobimetinib	Untreated melanoma with brain metastasis
NCT04721132	II	Recruiting	Bevacizumab	Atezolizumab	–	Resectable liver cancer
NCT02921269	II	Completed	Bevacizumab	Atezolizumab	–	Recurrent, persistent, or metastatic cervical cancer
NCT03141684	II	Recruiting	Bevacizumab	Atezolizumab		Advanced unresectable alveolar soft part sarcoma
NCT01950390	II	Active	Bevacizumab	Ipilimumab	–	Stage III-IV melanoma
NCT04981509	II	Recruiting	Bevacizumab, Erlotinib	Atezolizumab	–	Advanced stage kidney cancer
NCT05211323	II	Recruiting	Bevacizumab	Atezolizumab	Gemcitabine, cisplatin	Advanced unresectable liver cancer
NCT02997228	III	Recruiting	Bevacizumab	Atezolizumab	Combination chemotherapy	Mismatch repair deficient, metastatic, colorectal cancer
NCT02853318	II	Completed	Bevacizumab	Pembrolizumab	Low-dose cyclophosphamide	Recurrent ovarian, fallopian tube, or primary peritoneal cancer
NCT05468359	I/II	Recruiting	Bevacizumab	Atezolizumab	Cyclophosphamide, sorafenib	Pediatric solid tumors
NCT03396926	II	Active	Bevacizumab	Pembrolizumab	Capecitabine	Locally advanced, metastatic, or nonresectable microsatellite stable colorectal cancer
NCT03172754	I/II	Recruiting	Axitinib	Nivolumab	–	Advanced renal cell carcinoma
NCT04996823	II	Recruiting	Axitinib	Ipilimumab	–	Advanced melanoma
NCT04338269	III	Active	Cabozantinib	Atezolizumab	–	Inoperable, locally advanced, or metastatic renal cell carcinoma
NCT05805501	II	Recruiting	Axitinib	Triagolumab, Tobemstomig, Pembrolizumab	–	Previously untreated, unresectable locally advanced or metastatic clear-cell renal cell carcinoma
NCT04493203	II	Recruiting	Axitinib	Nivolumab	–	Unresectable stage III or IV melanoma
NCT02133742	Ib	Completed	Axitinib	Pembrolizumab	–	Advanced renal cell cancer
NCT04919629	II	Recruiting	Bevacizumab	Pembrolizumab	APL-2	Recurrent ovarian, fallopian tube or primary peritoneal cancer
NCT05231122	II	Recruiting	Bevacizumab	Pembrolizumab, anti-CD40 CDX-1140	–	Recurrent ovarian cancer
NCT02636725	II	Completed	Axitinib	Pembrolizumab	–	Advanced alveolar soft part sarcoma and soft tissue sarcomas
NCT04370509	II	Recruiting	Axitinib	Pembrolizumab	–	Locally advanced metastatic clear cell kidney cancer
NCT03092856	II	Active	Axitinib	Anti-OX40	–	Metastatic kidney cancer

## Regulation of tumor angiogenesis by myeloid cells

The vasculature is the avenue through which circulation-derived immune cells are recruited into the solid tumors. The infiltrating immune cells are exposed to the local vascular niche and interact with ECs mainly through paracrine mechanisms. The infiltrating immune cells locoregionally regulate vascularity, potentially modulating sprouting angiogenesis and vascular abnormalities. Here we discuss the regulatory mechanism underlying tumor angiogenesis by myeloid cells, which may serve as key therapeutic targets for vasculature-based cancer treatment.

**Fig. 2:**
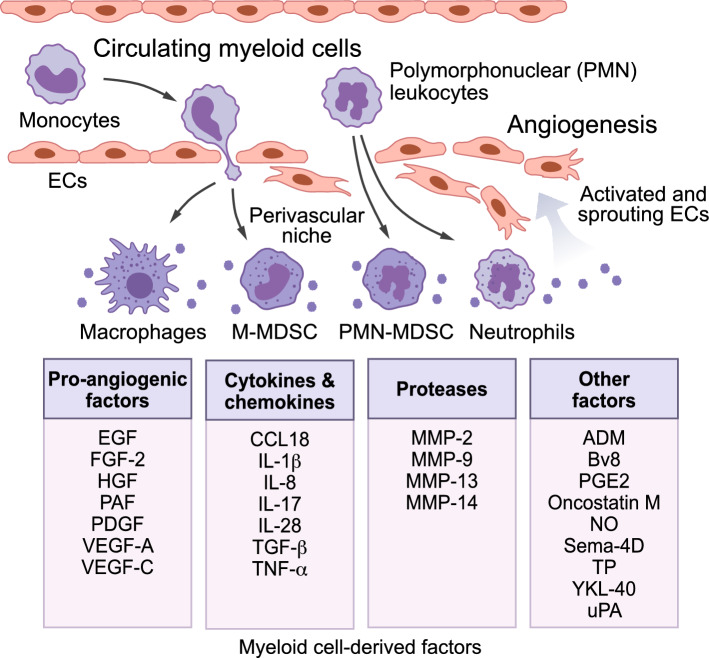
Regulation of tumor angiogenesis by myeloid cells. Tumor-infiltrating myeloid cells, including macrophages, MDSCs, and neutrophils, interact with ECs and modulate tumor angiogenesis through secreted factors

Tumor-infiltrating myeloid cells, mainly including macrophages, MDSCs and neutrophils, regulate tumor angiogenesis by secretion of a variety of pro-angiogenic factors. For instance, TAMs and MDSCs are known to secrete pro-angiogenic factors that stimulate EC proliferation and sprouting, leading to tumor angiogenesis and progression [98–100].Neutrophils serve as an additional source of released pro-angiogenic factors that regulate tumor growth and metastasis [101–103]. Myeloid cells can also indirectly enhance tumor angiogenesis by expressing matrix proteases and mesenchymal-associated factors that facilitate EC migration and vascular remodeling and maturation. In addition, myeloid cells, particularly perivascular macrophages, also contribute to dynamic vascular permeability in tumor [104]. Therefore, myeloid cells can regulate tumor angiogenesis through both direct secretion of pro-angiogenic factors and indirect modulation of the TME with multiple mechanisms potentially involved (Fig. 2).

### Macrophages

#### Macrophage-produced pro-angiogenic factors

Macrophages are a major cellular component of solid tumors [105]. TAMs promote tumor angiogenesis by secreting a plethora of pro-angiogenic growth factors, cytokines, and chemokines that induce EC proliferation and migration, including EGF[106], FGF-2/bFGF [107], platelet-activating factor (PAF) [108], PDGF [109], VEGF [110–112], TNF-α [113], IL-1 [114, 115], IL-8/CXCL8 [116, 117], and CCL18 [118]. TAMs undergo alternative polarization in the TME to stimulate tumor angiogenesis [107, 119], which is characterized by elevated expression of these pro-angiogenic factors [120]. Macrophages are a major source of pro-angiogenic factors, particularly VEGF, that are present in tumors. Macrophages employ diverse mechanisms to express VEGF, mainly induced by hypoxia through HIFs-mediated transcriptional activation and further stimulated by multiple cytokines like IL-1β [121–123] and CCL18 [118]. Moreover, TAMs significantly contribute to production of proteases, particularly matrix metalloproteinase (MMP)-9, presented in the TME [124], which directly facilitates EC overgrowth and sprouting by remolding ECM and indirectly activates ECs by providing the active form of VEGF as a result of cleaving VEGF and releasing it from the binding to ECM [125–129]. Bone marrow-derived, MMP-9-expressing macrophages also participate in tumor neovascularization together with vascular endothelial progenitor cells [130], providing an additional mechanism for tumor angiogenesis. TAMs also release various additional factors that have pro-angiogenic activity, such as adrenomedullin (ADM), PGE2, Sema-4D, thymidine phosphorylase (TP), urokinase-type plasminogen activator (uPA), and YKL-40. For instance, ADM induces EC proliferation and tumor angiogenesis and growth [131], PGE2 enhances EC motility and survival, contributing to tumor angiogenesis [132], Sema-4D binds to its receptor Plexin-B1 on ECs to induce tumor angiogenesis [133], TP stimulates EC migration [134], uPA promotes ECM degradation and vascular invasion [135], and YKL-40 activates MAPK signaling in ECs, leading to increased expression of VEGFR-2 that facilitates vessel sprouting [136]. Targeting tumor macrophage-released pro-angiogenic factors represent a promising strategy for therapeutic modulation of tumor angiogenesis.

#### Perivascular macrophages

Macrophages expressing Tie2 receptor (also known as Tek) often reside near vasculature and exhibit high angiogenic potential, playing a significant role in physiological and pathological angiogenesis [137]. A subpopulation of Tie2^+^ macrophages show a pro-angiogenic activity during embryogenesis [138]. These Tie2^+^ tissue macrophages release VEGF-C and soluble VEGFR1 to bridge between EC tip cells and modulate vessel branching in development [139, 140]. Similar to these findings, bone marrow-derived TAMs cluster around tumor vasculature and co-express Tie2 and CD206, showing more robust pro-angiogenic activity than Tie2^–^ monocytes or macrophages [141, 142]. Tie2^+^ monocytes express a number of tumor-promoting genes including *Mmp9, Vegfa, Cxcl12, Tlr4, Nrp1, and Pdgfb* at a high level [143], and their pro-angiogenic potential could be further stimulated by EC-derived factors in the perivascular niche [144]. The presence of Tie2^+^ macrophages in tumor regions has been linked to increased tumor microvascular density, enhanced tumor grade and distant metastasis, and reduced survival rates in human patients [145–147]. Perivascular macrophages accumulate in the tumor microenvironment following chemotherapy, radiotherapy, and anti-angiogenic therapy, contributing to vascular reconstruction, and potentially leading to tumor relapse [112, 148–151]. These macrophages originate from the precursors of a subset of Tie2^+^ circulating monocytes and are attracted to tumors by chemotaxis, induced by EC-derived angiopoietin-2 (ANG-2), a ligand of Tie2 [141, 144, 152]. Interaction with ECs stimulates Tie2 expression in TAMs and enhances the production of pro-angiogenic factors by these macrophages [144]. Ang-2 also drives Tie2^+^ macrophages to express IL-10 and CCL17, which inhibit T-cell proliferation and disrupt vascular homeostasis [153].

Recent studies have shed light on the importance of perivascular macrophages in the TME. In addition to promoting angiogenesis, these macrophages, residing near blood vessels, promote the formation of the vascular niche that contributes to tumor progression. Activation of these macrophages by extracellular matrix proteins, such as TNC (tenascin-C), through toll-like receptor 4 (TLR4) signaling, leads to the secretion of nitric oxide (NO) and TNF-α [154]. These factors, in turn, induce the expression of niche components in ECs, facilitating the establishment of a supportive TME for tumor growth and metastasis. These macrophages usually acquire anti-inflammatory phenotypes, contributing to spatially restrict immunosuppression in the vascular niche. As such, tumor EC-derived IL-6 induces alternative polarization and immunosuppressive phenotypes in perivascular macrophages [155]**.** In addition, Lyve-1^+^ macrophages have a critical role in creating a pro-angiogenic TME through maintaining and expanding a perivascular mesenchymal cell population, ultimately establishing a specialized niche that supports tumor progression [156]. Macrophage-derived TNF-α and endothelial TNF receptor are identified as crucial components of this regulatory mechanism. Perivascular macrophages, activated via TNC and TLR4 to induce the formation of pro-tumor vascular niche that drives tumor metastasis [154]. The spatial interaction between macrophages and ECs provides strong evidence for the intricate crosstalk that stimulates angiogenesis and tumor progression, metastasis, and therapy resistance [109].

#### Macrophage-mediated vascular maturation

Macrophages regulate vascular maturation under physiological and pathological conditions. As a resident macrophage population in brain, microglia maintain the integrity of blood–brain barrier that mainly consist of tightly associated ECs [157, 158]. Loss of NG2 proteoglycan in myeloid-specific and pericyte-specific cells leads to significant reductions in early-stage intracranial tumor growth [159, 160]. Myeloid-specific NG2 loss-induced vascular deficits, characterized by poor pericyte coverage on ECs and immature vessel, result in smaller vessel diameter, lower patency, increased leakiness, inefficient blood flow in tumor vasculature, and elevated intratumoral hypoxia [159]. TAMs promote pericyte coverage and stabilize tumor vasculature through the secretion of PDGF-B, contributing to vascular maturation [161]. Adenosine deaminase 2 (CECR1) is highly expressed by TAMs, contributing to tumor angiogenesis [161]. Increased CECR1 expression correlates with higher microvascular density in GBM tissues. Inhibition of CECR1 reduces new vessel formation, while CECR1 stimulation promotes vascular maturation through paracrine activation of pericytes via PDGFB-PDGFRβ signaling [161].

#### Macrophage-mediated vascular permeability

VEGF was originally identified as vascular permeability factor (VPF) as a result of its potent ability to enhance vessel permeability, resulting in vascular leakage [162]. TAM-derived VEGF-A may, therefore, induce local vascular permeability in tumors. Consistent with this hypothesis, real-time intravital imaging reveals that dynamic vascular permeability occurs concurrently with cancer cell invasion and Tie2^+^ macrophage infiltration in the perivascular niche [163]. Genetic deletion of VEGF in TAMs reverses vascular permeability and cancer cell intravasation [163], suggesting a role of TAMs for regulation of vascular permeability. TAMs regulate vascular permeability through VEGF-induced downregulation of vascular junction proteins ZO-1 and VE-cadherin and through VLA4-mediated disruption of vascular adhesion proteins VCAM1 in ECs [163, 164]. In addition, M2-like polarized macrophage-derived exosomes containing miR-23a, miR-155 and miR-221 induces angiogenesis and vessel leakiness [165, 166], serving as an alternative mechanism for regulating tumor vascular permeability.

### MDSCs

MDSCs are pathologically activated granulocytes (granulocytic or polymorphonuclear MDSCs, PMN-MDSCs) and monocytes (monocytic MDSCs, M-MDSCs) with potent immunosuppressive activity [167, 168]. MDSCs regulate immune responses in physiological and pathological conditions, including pregnancy, cancer, chronic infection, sepsis and autoimmunity [169]. In addition to their well-established role for direct suppression of lymphocyte activity, MDSCs secrete various pro-angiogenic molecules to induce tumor angiogenesis [100, 170, 171]. Tumor-associated Gr1^+^CD11b^+^ mouse MDSCs produce MMP-9 and release VEGF-A to promote angiogenesis [172]. Consistent with these findings, tumor-infiltrating MDSCs express MMP-2,-13,-14 at a high level [173], and overexpression of MMP inhibitor TIMP-2 reduces MDSC infiltration and vascular density in tumor [174], suggesting a critical role of protease for MDSC-mediated tumor angiogenesis. Moreover, G-CSF stimulates Stat3-dependent MDSC expression of Bv8 [175], a potent driver of myeloid cell-dependent tumor angiogenesis [176]. MDSCs also express FGF-2 [171], PDGF [177], IL-1β [178], IL-28/IFN-λ [179, 180], TGFβ, EGF, and HGF [181] that can stimulate EC proliferation and migration, contributing to tumor angiogenesis [182]. In addition, MDSCs could directly differentiate into ECs [172] and induce tumor cell formation of vascular mimicry (VM) [183], serving as alternative processes to sprouting angiogenesis.

### Neutrophils

Neutrophils are the most abundant innate immune cells in bone marrow and peripheral blood [184]. Neutrophils have emerged as an important component of the TME, but their functional role in cancer is still controversial [185]. In accordance with their critical functions in developmental angiogenesis [186, 187], neutrophils modulate tumor angiogenesis by providing pro-angiogenic factors in a time- and tumor context-dependent manner, contributing to tumor growth and metastasis [101, 102]. Tumor-associated neutrophils secrete a plethora of pro-angiogenic molecules including VEGF [188, 189], FGF-2 [190], Bv8 [191, 192], IL-17 [193], and MMP-9 [188, 194]. Neutrophil-derived oncostatin M also up-regulates the secretion of VEGF [195], and reprograms and degrades the ECM which then primes the environment for angiogenesis [195]. MMP-9 released by neutrophils promotes the activation of VEGF and subsequent angiogenesis and tumor progression [129, 194, 196]. Neutrophils also carry an intracellular pool of VEGF and mediate its rapid secretion [197]. Interestingly, IFN-β inhibits the infiltration of proangiogenic neutrophils that express VEGF, MMP-9, and CXCR4 and reduces tumor growth, suggesting a potential therapeutic approach for targeting neutrophil-mediated tumor angiogenesis [188]. These findings together suggest that neutrophils support the pro-angiogenic switch during cancer development [194]. As such, neutrophils display different states based on the expression of markers such as HIF-1α, arginase 1, and MMP-9, in which HIF1α^+^ neutrophils significantly correlate with greater angiogenesis and worse overall survival [198]. In addition to their role in directly driving pro-angiogenic functions, neutrophils can also indirectly promote angiogenesis by activating pro-angiogenic functions of other immune cells [103]. For example, neutrophils reprogram T cells to acquire regulatory-like phenotypes and support their expression of IL-10, IL-17, and VEGF to promote angiogenesis [199].

Neutrophils also contribute to tumor vascularization through several non-angiogenic mechanisms, such as neutrophil extracellular trap (NET) formation [174, 175], vessel co-option, and VM mechanisms [200, 201]. NETs, the release of web-like DNA structures, constitute an important mechanism by which neutrophils prevent pathogen dissemination or deal with microorganisms of larger size [202]. Cancer cells can induce NET formation by neutrophils, leading to tumor angiogenesis [203, 204]. NET-associated myeloperoxidase produces H_2_O_2_ released to ECM and activates NF-κB-mediated signaling in ECs, resulting in enhanced EC proliferation and migration [205]. Angiopoietins (ANG-1/-2) also induce NETs formation and promote neutrophil adhesion to endothelium and stimulated EC proliferation [206]. Finally, VM structures provide vascular channels for neutrophil infiltration and activation, leading to their expression of arginase, CCL2, CXCR4, and MMP-9 to promote angiogenesis and evade anti-angiogenic therapy [201], collectively suggest a critical role of neutrophils for tumor angiogenesis.

## Conclusion remarks and future perspectives

Anti-angiogenesis, vessel normalization, and endothelial reprogramming stand out as promising strategies for targeting the vasculature in cancer treatment. They hold significant potential when combined with various anti-cancer approaches including, but not limited to, radiotherapy, chemotherapy, molecular targeted therapy, and immunotherapy. The application of these strategies in clinical settings might require optimization based on factors like tumor type, size, stage, location, and pathology to achieve the maximal efficacy in combination therapy. Particularly, genetic, epigenetic and metabolic reprogramming of tumor ECs may offer unique opportunities to empower T cell-based immunotherapy, considering that endothelial reprogramming could (1) inhibit excessive angiogenesis and suppress vascular aberrancy, leading to increase vessel delivery function to improve lymphocyte infiltration as well as to relieve intratumoral hypoxia and to activate these lymphocytes, (2) regulate adhesion molecule expression on ECs to promote lymphocyte attachment to endothelium and their recruitment to the tumors, and (3) reverse pro-tumor immunity that is induced by locally EC-derived immunosuppressive molecules, facilitating lymphocyte activation.

Among these innovative strategies, the induction of HEV neogenesis has emerged as a promising strategy to augment anti-tumor immunity and vessel functionality. HEVs play a vital role in lymphocyte trafficking and activation, serving as a critical target for therapeutic modulation of immunocyte infiltration [207]. Recent single-cell RNA-seq analyses suggest their significant involvement in upregulated expression of EC activation markers and co-stimulatory molecules that regulate dendritic cell function and T cell activation [208]. Activation of lymphotoxin β receptor (LTβR) signaling induces the formation of HEVs and T cell activation, and thereby sensitizes tumors to anti-angiogenic and anti-PD-L1 therapy [209], collectively suggesting that better understanding the immunostimulatory functions of HEVs may open new avenues for immunotherapeutic interventions in the future.

Despite these advancements, there are still several major limitations and challenges that restrict vascular-targeting therapy in cancer, due to treatment toxicity, vascular heterogeneity in cancer, and the lack of reliable biomarkers hinder the effectiveness and applicability of current treatments. Notably, tumors can develop resistance to traditional anti-angiogenic treatments by compensatory activation of additional pro-angiogenic pathways to sustain tumor vascularization and by activating HIF-1α to support tumor growth and progression in low-oxygen conditions. One particular challenge is to develop a vascular-targeting strategy to selectively promote the infiltration of cytotoxic T cells and/or NK cells, but not immunosuppressive myeloid cells. Future spatiotemporal analysis of tumor specimens at single-cell transcriptome, epigenome, and metabolome levels will help address these challenges, as they need a deep understanding of the complex interplay between tumor vasculature and immune system in the tumor microenvironment.

Further research into the interaction of infiltrating myeloid cells with ECs regulates tumor angiogenesis, providing insights into the resolution of vascular formation, maturation, and aberrancy in cancer. Understanding of the underlying regulatory mechanism for tumor angiogenesis may lead to identification of new therapeutic targets for anti-vascular therapy, contributing to development of more efficient approaches for anti-angiogenesis, vessel normalization, and endothelial reprogramming therapy. It also remains largely unclear how ECs spatiotemporally regulate the immunity of these macrophages, MDSCs, and neutrophils in the vascular niche. New knowledge filling this gap may help understand tumor immunosuppression and lead to development of new myeloid cell-based immunotherapy for cancer treatment.

## Data Availability

Data sharing is not applicable to this article as no datasets were generated or analyzed for this review article.
